# The Launch of the International Society for Children’s Environmental Health and the Environment (ISCHE)

**DOI:** 10.1289/ehp.1104435

**Published:** 2011-10-01

**Authors:** David C. Bellinger, Lynn R. Goldman, Bruce P. Lanphear, Brenda Eskenazi, David E. Jacobs, Mark Miller,

**Affiliations:** D.C.B. is Professor of Neurology, Harvard Medical School, and Professor in the Department of Environmental Health, Harvard School of Public Health. E-mail: David.Bellinger@childrens.harvard.edu; L.R.G. is Dean, George Washington University School of Public Health and Health Services, and Professor, Environmental and Occupational Health and Pediatrics, Children’s National Medical Center.; B.P.L. is Senior Scientist of Child & Family Research Institute, BC Children’s Hospital and Professor in the Faculty of Health Sciences, Simon Fraser University.; B.E. is the Jennifer and Brian Maxwell Professor of Maternal and Child Health and Epidemiology and Director of the Center for Environmental Research and Children’s Health (CERCH), School of Public Health, University of California at Berkeley.; D.E.J. is Director of Research, National Center for Healthy Housing.; M.M. is the director of the University of California, San Francisco, Pediatric Environmental Health Specialty Unit and a Public Health Medical Officer for the California Environmental Protection Agency.

Children’s environmental health issues are inherently multidisciplinary, often requiring integration of the skills of epidemiologists, analytical chemists, toxicologists, social scientists, clinicians, public health practitioners, policy makers, specialists in living and learning environments (e.g., housing, schools), and community leaders, among others. The diverse group of professionals whose primary interests concern children’s environmental health research and policy has lacked a professional home that facilitates interdisciplinary collaborations. Many scientific and medical societies include children or the environment among the topics on which members focus, but rarely have the two been combined in a way that provides an international forum dedicated to discovering ways to protect children from environmental hazards.

To address the need for a professional society that provides such a forum, a new organization was recently formed and incorporated, the International Society for Children’s Health and the Environment, or ISCHE (http://www.ische.ca). The first election of officers was held in April 2010.

The goals of ISCHE reflect the broad array of issues germane, worldwide, to children’s environmental health and safety:

To translate research into policies that protect children’s health by enhancing the quality of their environments
To catalyze the development of multidisciplinary research aimed at protecting children from environmental hazards
To enhance the training of professionals in children’s environmental health
To promote the delivery of services to create healthy environments for children
To enhance surveillance of childhood exposures and environmentally induced diseases or disabilities
To enhance clinical care of children with environmentally induced disease or disability.

Because of its multidisciplinary membership, ISCHE is well positioned to make current scientific findings more accessible to the health care, public health, and policy communities via position papers, technical reports, and testimony. We envision this happening in several ways, including the following:

ISCHE will foster interdisciplinary collaborations, providing an environment in which individuals from the many disciplines involved in children’s health and environments can come together to share information and to combine forces to work on topics of common interest.
ISCHE will connect individuals or organizations seeking expertise on children’s health and the environment with members who might be able to serve as speakers, reviewers, or consultants.
ISCHE committees will review and translate research to promote evidence-based policies, standards, and educational initiatives that protect children from adverse environmental influences and promote healthier environments. These committees will also propose critically needed children’s health research and facilitate pooled analyses. The committees will be composed of a standing group of scientists drawn from the ISCHE membership, as well as invited scientists who bring expertise on a specific issue. The conclusions and policy recommendations of these committees will be published in the scientific and medical literature. On occasion, committees might also produce op-ed pieces for the lay press on topical issues.
ISCHE will help to establish children’s environmental health as an essential part of the curricula in various health training programs internationally and assist in the creation and advancement of appropriate research and policy initiatives for developing as well as developed nations.

“International” is the first word in the title of ISCHE because we believe that children’s environmental health and safety must be viewed from a global perspective. Some recent incidents involving children’s harm from environmental chemical exposures have occurred in developing countries (e.g., the recent epidemic of severe childhood lead poisoning in Nigeria) and countries in transition (e.g., melamine in infant formula in China), but others have occurred in highly industrialized countries (e.g., the Fukushima Daiichi nuclear disaster in Japan). To contribute to capacity building worldwide, membership dues are reduced for individuals from developing countries (and waived for students from these countries).

Eventually ISCHE plans to hold an annual membership meeting and research conference. However, during this formative time, we are coordinating our meetings with those of other societies. For example, in September 2011, ISCHE met at the International Society for Environmental Epidemiology (ISEE) meeting in Barcelona, Spain. In addition to holding meetings for its officers and members, ISCHE sponsored a pre-meeting Workshop on Assessment of Neurobehavioral Effects of Environmental Exposures on Children. This workshop was intended to inform attendees of the methodologic issues pertinent to the design, conduct, and analysis of studies on associations between children’s environmental exposures and their neurodevelopment. In future years, at meetings of ISEE and/or other societies, ISCHE will organize additional workshops, focusing on specific exposures (e.g., pesticides, bisphenol A), target organ systems (e.g., the lung), methods (e.g., genome-wide association studies), and policies.

ISCHE is dedicated to providing a professional home for those with scientific, policy analysis, educational, and clinical interests in children’s environmental health. ISCHE plans to work with other organizations with related missions, both in the United States and internationally, to ensure complementarity of our efforts rather than duplication.

